# Bivariate Gaussian bridges: directional factorization of diffusion in Brownian bridge models

**DOI:** 10.1186/2051-3933-2-5

**Published:** 2014-03-01

**Authors:** Bart Kranstauber, Kamran Safi, Frederic Bartumeus

**Affiliations:** Department for Migration and Immuno-ecology, Max Planck Institute for Ornithology, Radolfzell, Germany; Department of Biology, University of Konstanz, Konstanz, Germany; ICREA-Movement Ecology Lab (CEAB-CSIC), Girona, Spain; Centre for Ecological Research and Forestry Applications (CREAF), Barcelona, Spain

**Keywords:** Dynamic Bivariate Gaussian bridge, Dynamic Brownian bridge movement model, Utilisation distribution, Animal tracking, GPS, Home range and space use modelling

## Abstract

**Background:**

In recent years high resolution animal tracking data has become the standard in movement ecology. The Brownian Bridge Movement Model (BBMM) is a widely adopted approach to describe animal space use from such high resolution tracks. One of the underlying assumptions of the BBMM is isotropic diffusive motion between consecutive locations, i.e. invariant with respect to the direction.

Here we propose to relax this often unrealistic assumption by separating the Brownian motion variance into two directional components, one parallel and one orthogonal to the direction of the motion.

**Results:**

Our new model, the Bivariate Gaussian bridge (BGB), tracks movement heterogeneity across time. Using the BGB and identifying directed and non-directed movement within a trajectory resulted in more accurate utilisation distributions compared to dynamic Brownian bridges, especially for trajectories with a non-isotropic diffusion, such as directed movement or Lévy like movements. We evaluated our model with simulated trajectories and observed tracks, demonstrating that the improvement of our model scales with the directional correlation of a correlated random walk.

**Conclusion:**

We find that many of the animal trajectories do not adhere to the assumptions of the BBMM. The proposed model improves accuracy when describing the space use both in simulated correlated random walks as well as observed animal tracks. Our novel approach is implemented and available within the “move” package for R.

**Electronic supplementary material:**

The online version of this article (doi:10.1186/2051-3933-2-5) contains supplementary material, which is available to authorized users.

## Background

The availability of global positioning system (GPS) and affordable satellite telemetry has revolutionised the study of animal movement, allowing users to estimate the location of individuals at high spatial and temporal resolution. Consequently, the amount of highly-resolved data has increased by orders of magnitude providing potential improvements and new challenges for the analysis of animal movement. For many applications, among others habitat and home range analysis, it is important to convert these trajectories into spatially explicit probability distributions. Previously, methods for estimating animal space use, such as minimum convex polygons (MCP) or kernel home range estimates, ignored the temporal sequence of the locations and instead concerned with the statistical independence of successive locations of the tracked animals. Now however, novel approaches can take profit from richer data sets by incorporating time (i.e. temporal autocorrelation) into the modelling of space use.

Brownian bridge movement models (BBMM) estimate space use intensity by stochastically modelling the movement of animals between any two consecutive locations [1, 2]. Brownian bridges connect two consecutive locations by conditional Brownian random walks that start at a given location and end at the following location with a duration equal to the observed time lag between the two locations. Brownian random walks are one of the simplest random walk models and assume movement in any direction to be equally likely, movement steps are drawn from a bivariate Gaussian distribution [3]. The interpolation, or bridge, incorporates both an empirically derived tendency to diffuse and an estimation of the error associated to the true locations. The BBMM has been extended to account for changes in the movement behaviour of animals (dynamic Brownian Bridge movement models: dBBMM [4]) by allowing the diffusion parameter of the Brownian motion to change according to changes in the behaviour of the animal along its trajectory [5, 6].

Brownian bridges have the benefit of being efficiently calculated and can be fitted straightforwardly to existing trajectories. Further, these bridges can convert a set of locations into a well-defined and spatially-explicit probability density function that models space use intensity (i.e., a normalized and rasterized spatial probability distribution). Such a probability surface can easily be associated to the corresponding landscape information (e.g. [7, 8]). As in any modelling procedure, however, there are constraining assumptions, i.e. the movement between locations is assumed to be diffusive (normal diffusion) and isotropic. In its dynamic version (i.e., the dBBMM), the normal diffusion assumption is somewhat relaxed, as that the dispersion parameter is allowed to vary over time (different diffusion coefficients can be estimated), accounting for rather complex multi-scale, composite-Brownian type of motions [9, 10]. Nevertheless, the movement between any two locations is equally likely to happen in all directions and is quantified as an isotropic diffusive process.

Directional bias has been incorporated differently in widely different movement models (e.g., correlated random walks, biased random walks, multi-scale random walks) (e.g. [11, 12]). Benhamou [13] introduced the idea of directional bias in bridging models. Biased Random Bridges [13] rely on the advection-diffusion model, which shows both a diffusive and an advective component, and can generate long-term drift. Essentially Benhamou assumes that the role of anisotropy in generating a “direction in diffusion” is minor and that most of the effect should come from the advection. It happens that the advection component disappears in bridge calculations. Because of this, classical Brownian bridge formulations can be used within Biased Random Bridges, except for diffusion anisotropy. In biased random walks the emergence of diffusion anisotropy depends on different movement parameters that shape the turning angle distributions and can contribute to directional bias [14]. Given that in Biased Random Bridges the advection component is lost a relevant question remains: does taking diffusion anisotropy into consideration in bridge models improve space use estimations?

### Bivariate Gaussian bridges

Here we present a novel approach to modelling animal movement as a generalisation of the BBMM: the Bivariate Gaussian bridges (BGB), which allows us to factorize diffusion (i.e. Brownian variance estimates ()) in two elementary directions: the direction toward the next location and the direction orthogonal to it. In the case of the BBMM (and dBBMM) the probability density around the mean position of the animal between any two locations is assumed to be isotropic. In BGB, however, we factorize the movement variance in a parallel and an orthogonal component to the straight line (with constant speed) connecting two consecutive locations (a segment). This results in two normally distributed probability densities Pr_*p*_ and Pr_*o*_.

Finally, all observation techniques have an error associated with estimating the true position of the animal. We add this location error distribution, which, for the sake of simplicity is assumed to be a bivariate normal distribution (standard deviation *δ*), to the probabilities Pr_*p*_ and Pr_*o*_ to account for this measurement error.

Like BBMM, BGB has the same benefit of a straightforward fitting of movement to empirical trajectories, and has the potential to more accurately capture directional correlation. It also allows the computation of an index of directionality (*I*_*d*_):


The index can vary between −1 and 1, where 0 means *σ*_*m*,*p*_ and *σ*_*m*,*o*_ are equal in size corresponding to Brownian motion. In case *I*_*d*_→1 there is no orthogonal diffusion an thus all movement is along the straight line, whereas *I*_*d*_→−1 indicates there is no parallel diffusion. Various studies have found turning angles to be informative measure for inferring behavioural state [15–17]. The correlation in heading has also been investigated in various contexts such as search efficiency [18] and orientation analysis [19] and has been measured by the sinuosity index [20]. We therefore think that an index capturing heading fluctuations of the animal between succssessive locations could be indeed a very informative measure of behaviour, that in combination with other measures could determine behavioural modes classification. For the variance estimation we used the dBBMM framework [4], accounting for changes in the diffusion variance across time. Hence BGB incorporate changes in the movement characteristics along long journeys and, since *I*_*d*_ can change continuously, provide additional information on changes in the directionality of the movement. Bivariate Gaussian bridge models should not only derive space use quantification more accurately (by estimating movement anisotropies), but should also allow to explore temporal patterns of *I*_*d*_, that can be used to elucidate changes in movement behaviour.

In the following, we formally introduce the BGB framework and assess its improvement (compared to the dBBMM) in predicting the locations of simulated correlated random walks with varying degrees of correlation and step lengths. Finally, we use a series of empirical trajectories of different animal species to assess how realistic the isotropy assumption is by investigation of *I*_*d*_ and comparing the estimated space use derived from the BGB (anisotropic) with the one derived from Brownian (isotropic) bridge movement models.

#### Variance estimation

**Figure 1:**
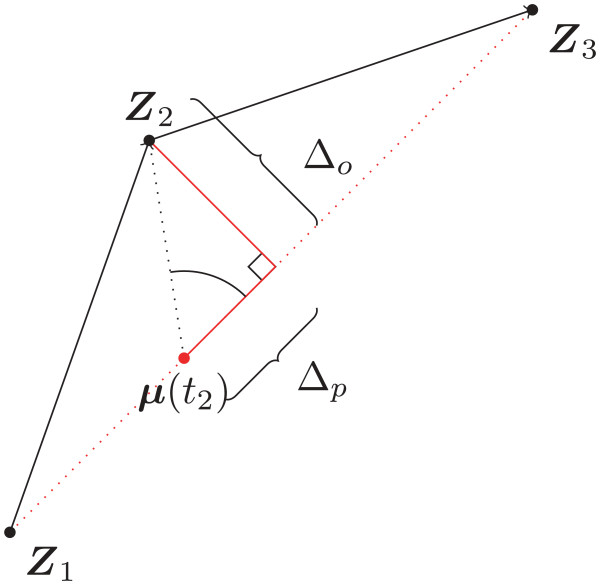
**An example of how the parallel and orthogonal deviation (** ***Δ***_***p***_ **and** ***Δ***_***o***_ **) from the expected location of** ***Z***_**2**_ **(** ***μ*** **(** ***t***_**2**_ **)) on the straight line connection can be derived for the segment between the location** ***Z***_**1**_ **and** ***Z***_**3**_ **.**

Both *σ*_*m*,*p*_ and *σ*_*m*,*o*_ are initially unknown and need to be estimated from the trajectories. Using a leave-one-out and a maximum likelihood approach [2], we estimate the most likely combination of *σ*_*m*,*p*_ and *σ*_*m*,*o*_ values by maximising the likelihood for the locations that were left out. For every second location the orthogonal and parallel distance (*Δ*_*p*_ and *Δ*_*o*_) to the expected location on the straight line connection between the previous and next location is calculated by projecting the vector from the expected location to the observed location onto the vector from the expected location to the next location. This procedure is illustrated in Figure 1. Using these distances the likelihood for *σ*_*m*,*p*_ and *σ*_*m*,*o*_ can be calculated and maximized.

Finally, since changes in the behaviour of tracked animals can lead to changes in magnitude and relative proportion of *σ*_*m*,*p*_ and *σ*_*m*,*o*_ over time, we allow the variance estimation in the BGB to dynamically take different values, resulting in dynamic Bivariate Gaussian bridges (dBGB). Using the Bayesian Information Criterion (BIC) in conjunction with estimation of variance within and between sections of the trajectory we identify significant changes in the composition of *σ*_*m*,*p*_ and *σ*_*m*,*o*_ as introduced by Kranstauber et al. [4] based on the work of Gurarie et al. [17]. This allows the model to track changes in a trajectory along both, scale of movement and in the directionality. The algorithm uses a window of a set number of locations in which the best values for *σ*_*m*,*p*_ and *σ*_*m*,*o*_ are searched for. Within this window, both *σ*_*m*,*p*_ and *σ*_*m*,*o*_ are calculated over the entire length of the window as well as for any combination of subsections before and after possible break points. A likelihood value is calculated for each description of the window that contains either no break, a single break in *σ*_*m*,*p*_, a single break in *σ*_*m*,*o*_, or a break in both. These likelihoods are then compared using the BIC. For each window we choose the optimal descriptor based on the BIC. The window is then moved 1 step through the trajectory and the likelihood optimization is repeated. For each segment we thus obtain multiple estimates of *σ*_*m*,*p*_ and *σ*_*m*,*o*_ that are averaged using the variances. For the dBGB case, *σ*_*m*,*p*_ and *σ*_*m*,*o*_ are allowed to change independently. This allows in total for 4 different change scenarios: no change, a change in either *σ*_*m*,*p*_ or *σ*_*m*,*o*_, or a change in both. Both, the size of the window and the margin, are parameters of the algorithm that define the granularity of behavioural change detection in the dynamic estimation of *σ*_*m*,*p*_ and *σ*_*m*,*o*_ in dBGB, or *σ*_*m*_ in the dBBMM. Margins are the minimal number of locations used for variance estimation, and thus define the number of possible changes within the window. Larger windows have more power to identify changes in behaviour but come at the cost of not being able to describe frequent changes in behaviour. Since more variables (*σ*_*m*,*p*_ and *σ*_*m*,*o*_ vs. *σ*_*m*_) are involved, it may be sensible to use larger windows and margins for a more accurate approximation of the true values. It is important to explore various parameter setting and assess if the results make sense and describe the expected behavioural changes. One possible way to do this is by using cross-validations [4].

#### Utilisation density calculation

With *σ*_*m*,*p*_ and *σ*_*m*,*o*_ for every segment known, the probability of utilisation of an area (e.g., a map grid cell) is defined by the orthogonal (*Δ*_*o*_) and parallel distances (*Δ*_*p*_) from the grid cell center to the expected location on the connecting line of the segment using a normal distribution. To calculate the integrated probability density, each segment is split into equally sized integration steps proportional to the time interval between the two consecutive locations defining the segment. For each time step the expected location can be calculated, using this expected location *Δ*_*p*_ and *Δ*_*o*_ to each grid center can be calculated. These functions are implemented in R [21] and will be available within the move package [22]. For details we refer to the Methods Section at the end of the manuscript.

**Figure 2:**
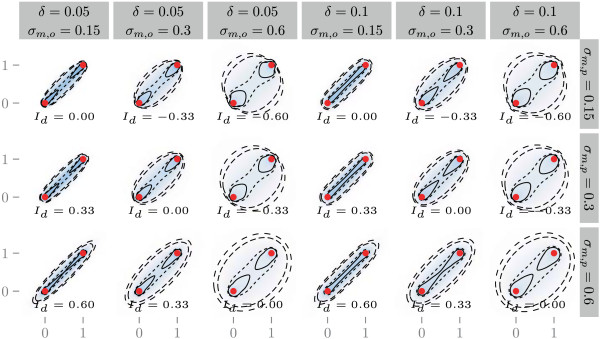
**A variety of Bivariate Gaussian bridges moving from (0,0) to (1,1).** The bridges are calculated using various location errors (*δ*), *σ*_*m*,*p*_ and *σ*_*m*,*o*_. The contours show the 0.3, 0.5, 0.9 and 0.95 UD contours. Colours indicates the space use density. The red points correspond to the start and end locations of the movement.

Figure 2 shows densities and contours of bridges using different parameter combinations. To optimize the calculation time, we restrict the estimation of probability for every numerical integration step to a bounding box with a size of 5 times the standard deviation around the expected location (*μ*). This saves computation time substantially by avoiding the need to estimate probabilities for the entire grid, including locations so far away that the probabilities are negligibly small. This accurately quantifies more than 99.99 percent of the cumulated utilisation probabilities, allowing, at an equal computational time, higher temporal and spatial resolution of the probability density calculation, thereby increasing overall efficiency with little loss of information (note that this optimization could also be used within the BBMM and the dBBMM).

### Validation

We evaluate the dBGB first on a set of simulated trajectories and then apply it to GPS tracks of a selection of different species.

#### Simulated trajectories

To explore the behaviour of *σ*_*m*,*p*_ and *σ*_*m*,*o*_, we simulated a variety of random trajectories using a correlated random walk function ([23] implemented by Calenge et al. [24]). We varied both the amount of correlation within the random walk from 0.5 to 0.999 as well as the scale of step lengths from 0.1 to 10. Each track consisted of a total of 249 (odd number required for the leave-one-out approach) locations whereby a circular Gaussian error with a standard deviation of 0.01 was added to each location.

The dBGB variances as well as the dBBMM variances were estimated on these trajectories. Since the simulated tracks did not contain any behavioural change, window and margin sizes, which define the granularity of behavioural change detection in the dynamic estimation of *σ*_*m*,*p*_ and *σ*_*m*,*o*_ in dBGB as well as *σ*_*m*_ in dBBMM, were chosen to be large (margin: 31 and window size: 71). In order to evaluate the performance of the estimation of the utilisation distribution (UD), we compared the predicted probability densities of the dBGB to those of the dBBMM for these trajectories. To test performance of each method we did cross validations by excluding 35 locations, that were regularly spaced in time. The UD was calculated using each method without the selected locations. We then examined the performance by extracting the UD values at the omitted locations and calculating an index of performance. The performance index was calculated as the geometric mean of the probability densities of dBGB divided by the probability densities of dBBMM (following [4]) where values greater than 1 indicated better performance of dBGB.

#### Observed trajectories

To investigate performance differences of UD estimation with dBGB compared to dBBMM on real trajectories, 7 individual trajectories of various species (Table 1) were used. The data were organized and standardized using http://www.movebank.org[25]. For the sake of representation, we focused on the first 1000 relocations of every track, for the stork (*Ciconia ciconia*) we omitted the first 1700 locations because the animal was initially stationary. First we investigated whether real animal trajectories adhered to the assumptions of Brownian motion by calculating an index of directionality *I*_*d*_ over time.

**Table 1:** **An overview of the different tracks for which we evaluated the dBGB model**

Species		***T***	***N***	***I***_***d***_ (>95 ***%*** )	***I***_***d***_ (<95 ***%*** )		***C***	Reference
Eurasian eagle-owl (*Bubo bubo*)	20.00	66.07	1000	0.73	0.00	0.34	1.09	
Fisher (indiv #1) (*Martes pennanti*)	2.07	9.64	1000	0.46	0.04	0.20	1.11	[5, 26]
Fisher (indiv #2) (*Martes pennanti*)	15.03	20.88	919	0.40	0.02	0.18	1.08	[5, 26]
Straw-coloured fruit bat (*Eidolon helvum*)	5.02	5.50	434	0.92	0.00	0.58	1.52	
Turkey vulture (*Cathartes aura*)	60.00	45.29	1000	0.61	0.03	0.33	1.81	[27]
Waved albatross (*Phoebastria irrorata*)	89.97	64.88	1000	0.78	0.03	0.45	1.16	[28, 29]
White stork (*Ciconia ciconia*)	5.03	8.30	1000	0.74	0.02	0.42	1.00	

: Median interval between relocations (mins); *T*: Tracking period (days); *N*: Number of locations; *I*_*d*_ (***>*** 95%): Proportion of segments where *I*_*d*_ is larger than the 95% interval; *I*_*d*_ (***<*** 95%): Proportion of segments where *I*_*d*_ is smaller than the 95% interval; : Median *I*_*d*_; *C*: Performance index.

The variances of the tracks were calculated using a window size of 39 and margin of 15. We also simulated 1000 Brownian motion tracks with 39 locations (same length as the window size), as a confidence interval for the directionality index. We then evaluated how many of the segments fell outside the expected 95% interval. If substantially more than 5% of the *I*_*d*_ values fall outside the confidence interval we interpreted this as an indication that the trajectory as a whole did not correspond to Brownian motion.

To compare the performance of dBGB against dBBMM also in real animal trajectories, we again calculated the performance index by omitting 50 locations, which were used for a cross-validation. For this calculation we used the same window size and margin of 39 and 15.

Finally, we present the track of one individual straw coloured flying fox (*Eidolon helvum*) to visually highlight the obtained UD contours from dBGB in comparison with those obtained from the dBBMM. This African fruit bat roosts in the colony during the day and moves in a very directed manner to individual fruiting trees to forage during the night. We excluded segments during daytime where no movement was recorded.

## Results

### Simulated trajectories

**Figure 3:**
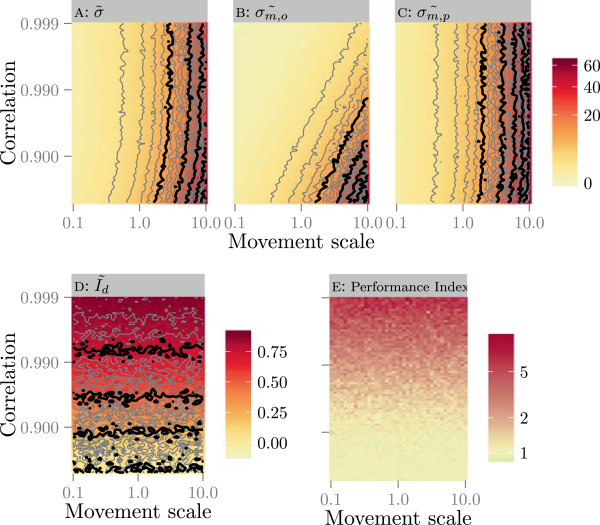
**The median values of** ***σ***_***m***_ **(A),** ***σ***_***m*,*o***_ **(B),** ***σ***_***m*,*p***_ **(C) and** ***I***_***d***_ **(D) and the performance index (E) in relation to the parameters of the simulated correlated random walks in separate panels.** The movement scales (i.e., step sizes) are indicated on the x axis while correlation of the turning angles is on the y axis. On the upper plots colour represents the variance values, while on the lower plots colour indicates *I*_*d*_ and the performance index. The lines represent isoclines as a visual guide for investigating the differences. Note that both axes are log transformed.

The correlated random walk simulations showed that with increasing movement scales (step sizes) both *σ*_*m*,*p*_ and *σ*_*m*,*o*_ increase (Figure 3B, C). In addition, the orthogonal standard deviation *σ*_*m*,*o*_ increased as the correlation of the random walk decreased. The Brownian motion standard deviation (Figure 3A) followed largely *σ*_*m*,*p*_ but was more influenced by a decrease in the correlation of the correlated random walk. The index of directionality *I*_*d*_ increased with increasing correlation but was not influenced by the movement scale (Figure 3D). Only in the region of both high correlation and small movement scales, *I*_*d*_ became scale dependent. This was due to the effect of the location error, shown in the Additional file 1 by repeating the same analysis on the same tracks with a higher location error. The performance index increased when *I*_*d*_ increased, at higher values for *I*_*d*_ (0.5 and up) the performance index doubled (or more) (Figure 3E). This means that the estimated UD associated to the locations omitted for the cross validation doubled.

### Observed trajectories

**Figure 4:**
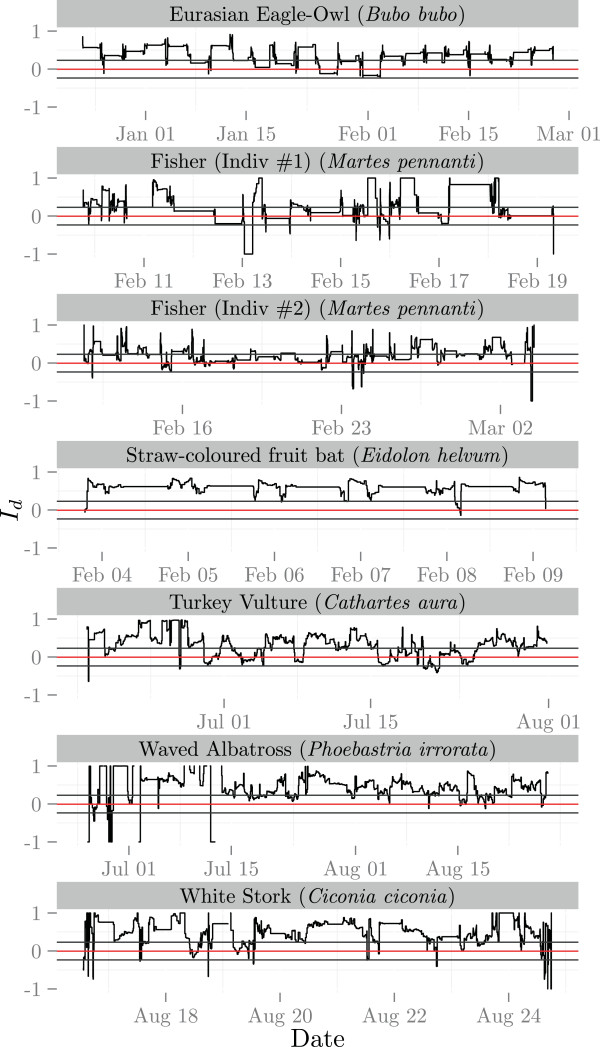
**Directionality index** ***I***_***d***_ **over time for different tracks.** The red and grey horizontal lines show the mean and 95% interval of the *I*_*d*_ for simulated Brownian random walks.

Investigating the variances of different tracks showed that large parts of the tracks deviated from what would be expected under a Brownian motion assumption. All 7 empirical trajectories in our study had a median *I*_*d*_ larger than 0 and a large proportion (on average 0.66) of the segments fell above the 95% percentile of the distribution of the simulated Brownian tracks (Table 1). If all tracks adhered to the assumption of Brownian motion, then we would expect that only 2.5*%* of the locations would fall above this interval. The index over time revealed that the tracks showed extended periods of time with a high *I*_*d*_ values interspersed by bursts of low *I*_*d*_ values (Figure 4). The cross validation on the trajectories showed that the dBGB resulted in either a similar or better fit compared to the UD derived from dBBMM (Table 1).

**Figure 5:**
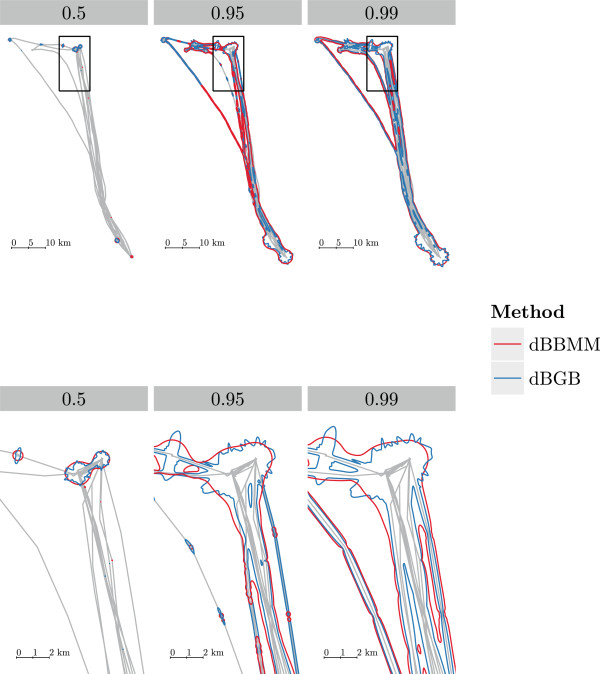
**Utilisation density (UD) contours (for 0.5, 0.95 and 0.99 space use probabilities) for one African fruit bat (** ***Eidolon helvum*** **).** The contours are narrower around the track for the dBGB, this is especially visible in the zoomed in section of the track. The grey lines show the track of the bat, the panels from left to right show the different UD contours.

Figure 5 shows the UD contours for several night tracks of an African fruit bat, including a zoomed section. The UD contours around the directed flights between the roost in the lower right corner and the foraging areas at the top of the plot are narrower. The contours around the foraging trees are very similar with the dBGB having a bit more well-defined areas.

## Discussion

Our method demonstrates the potential accuracy improvements when computing the UD, and highlights the constraints of initial Brownian bridge models [2] that assume isotropic and homogeneous diffusion across time. The method presented here relaxes both assumptions by: *i*) locally characterizing diffusion, thus becoming variable in time, and *ii*) analysing the diffusion properties across two major and orthogonal axes of motion, thus becoming variable in space. The directional bias in trajectories can be a product of various processes (e.g. correlated random walks, biased random walks, landscape features). Although we only formally investigate correlated random walks we think that dBGB performs equally well if the directional bias has a different cause because the variance estimates are adjusted to the observed trajectory.

Biased Random Bridges [13] assume an advective and a diffusive component, which can incorporate the effect on the directional bias attributable to diffusion anisotropy. More mechanistic insight can be extracted from a bridging model by decoupling advection from diffusion, and thus, Biased Random Bridges represent a clear conceptual improvement with respect to standard BBMM [2]. Nevertheless, Biased Random Bridges do not modify nor improve the estimated UD. Instead, BGB can approximate the idea of advection/diffusion by considering the diffusive non-isotropic process, and thereby improve the accuracy of space use and utilisation density distributions. We did not conduct a direct comparison between the dBGB and the Biased Random Bridges because for the latter no dynamic version is defined. It would be hard to assess where to attribute the differences to.

Worth noting are the jagged contours that appear when the directionality index (*I*_*d*_) is high, such as the high probabilities right in front and behind the observed locations. Within strongly directed movement periods these probabilities overlap with the previous and next segment. It is likely that these probabilities are visible in contour lines when an animal starts or stops a directed movement period, where the transition to a stop causes extension of the contours behind the observed stop location. These jagged contours can for example be observed in Figure 5 in the upper right corner where shuttling between the localities occurs. Other bridge models suffer from similar artifacts but they might be less conspicuous because the resulting contours are smoother. In any case, it is important to note, that despite these artefacts, UD estimates are overall more accurate than with former models, and that the previous models also contain biases even though perhaps less conspicuous.

In general, the dBGB predicts the location of the observed trajectories better and only in some cases performs slightly worse than the dBBMM. Better dBBMM performance over dBGB may be due to the fact that the estimation of more variables (*σ*_*m*,*p*_ and *σ*_*m*,*o*_ vs. *σ*_*m*_) increases the noise. The *I*_*d*_ provides a measure of directedness independent of the step sizes of the movement. It could also be used as an indication of where the largest differences between dBBMM and the dBGB are to be expected: essentially where the largest deviations of *I*_*d*_ from 0 are observed. When the time interval between observations increase, the directional persistence of the correlated random walk decreases [20]. We would therefore expect that *I*_*d*_→0 if the time interval between observations increase, this would mean the difference between the dBGB and dBBMM decreases. When the location error is of the same order of magnitude as the movement variances, the division between movement variance and location error becomes more difficult, resulting in estimates where one of the variances becomes 0. Also the directionality index (*I*_*d*_) becomes scale-dependent when location errors are high.

Although we do not investigate it here, it is likely that the estimated *I*_*d*_, *σ*_*m*,*p*_ and *σ*_*m*,*o*_ contain relevant intra and inter individual variation. For example, migrating versus sedentary herds of caribou have a far narrower turning angle distribution [30] which would result in a higher *I*_*d*_. Hence, studying *I*_*d*_, *σ*_*m*,*p*_, and *σ*_*m*,*o*_ spatiotemporal patterns across individuals, ecological contexts, or species, could provide more mechanistic insights into animal home range and space use behaviour.

## Conclusions

It is clear that many observed trajectories do not adhere to the assumptions of isotropic, homogeneous Brownian motion. Our model had the highest performance gain for correlated random walks with high directional correlation. Further work defining analytical descriptions of bridge functions for frequently used random walk models (e.g., correlated random walks, Lévy walks, or continuous-time random walks) is needed since the dBGB does not formally describe the probability density of any of these random walks.

## Methods

For notational convenience we followed the notation and variable definitions of Horne et al. [2].

### Probability density function

*Z*_*i*_ denotes the observed locations of the animal, at times *t*_*i*_ with a normal distributed observation error with standard deviation of *δ*_*i*_. *T*_*i*_ denotes the time gap between two observations and is calculated as *T*_*i*_=*t*_*i*+1_−*t*_*i*_. The expected center of the distribution of possible positions of the animal at time *t* in the time interval *t*_*i*_ till *t*_*i*+1_ is assumed to be as follows


The standard deviations are assumed to be independent in the orthogonal and parallel direction and increase between locations


In a first step, we transform the coordinates into a parallel and an orthogonal distance using Eq. 1 by projecting the vector ***μ***−***e*** on to ***μ***−***d***. This equation gives the parallel and orthogonal distances from ***μ*** to ***e*** when heading toward ***d*** from ***μ***.
1

The following equation defines the probability density function of the multivariate Gaussian distribution, where *k* is the number of dimensions, ***μ*** the center of the distribution and ***x*** a *k* dimensional vector for which the density is calculated.


Given we are working in two dimensions *k* is 2 and ***x***−***μ*** and ***Σ*** are defined as follows


where *z* is any location in the space.

Furthermore, if we assume the orthogonal and perpendicular distances to be uncorrelated, then *ρ*=0. The probability density function of the bivariate normal distribution is:


and can be simplified to:
2

#### Including location error

In order to include the locations errors we need to redefine our *σ* functions as,


### Likelihood

The multivariate log-likelihood is:


In order to estimate *σ*_*m*,*p*_ and *σ*_*m*,*o*_ by omitting every second location we have *i* in 1,3,5,…,*n*−1. We get the following set of equations:


Making the same assumption that there is no correlation between parallel and orthogonal variation (*ρ*=0) and filling out the log-likelihood equation we get


and simplify this to:


## Availability of supporting data

The source code for the methods are available in the R package “move”.

## Electronic supplementary material

Additional file 1: **Correlated random walk analysis with increased location error.** (PDF 316 KB)
