# Supplementary material for: Bivariate Gaussian bridges: directional factorization of diffusion in Brownian bridge models
Source: Mov Ecol. 2014 Mar 1;2(1):5. doi: 10.1186/2051-3933-2-5 (PMC4416317; doi:10.1186/2051-3933-2-5)
Supplement: Supplementary file 1 — Additional file 1: Correlated random walk analysis with increased location error. (PDF 316 KB) [file 40462_2013_32_MOESM1_ESM.pdf]

The performance of the dBGB on correlated random walks with increased location errors

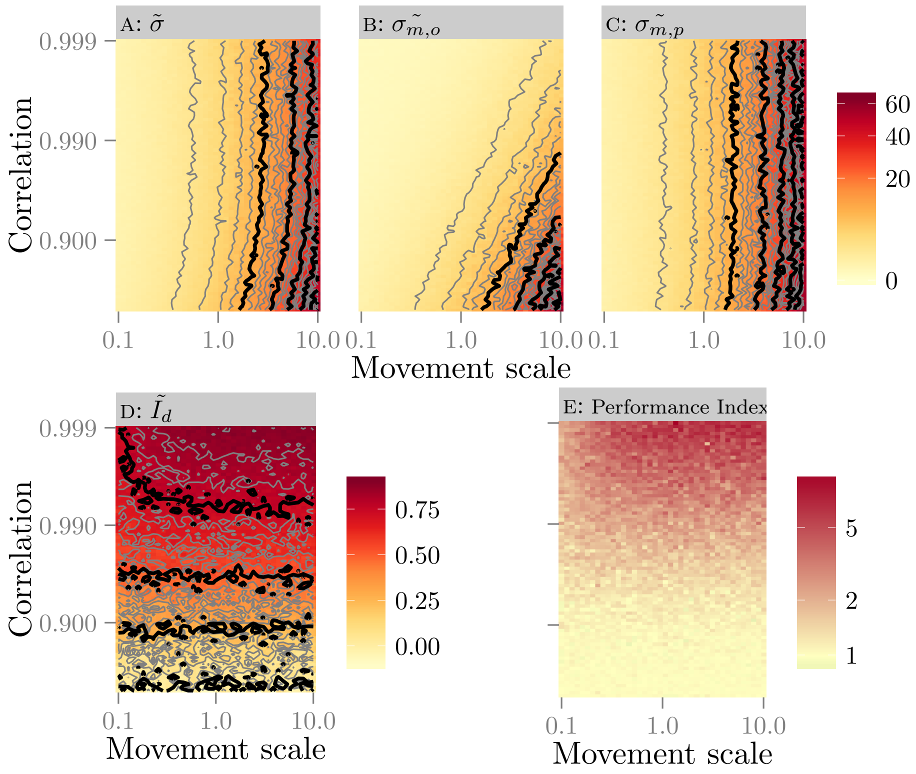

Figure 1: Repetition of the analyses with a higher location error (0.05) showing the effect on the directionality index.

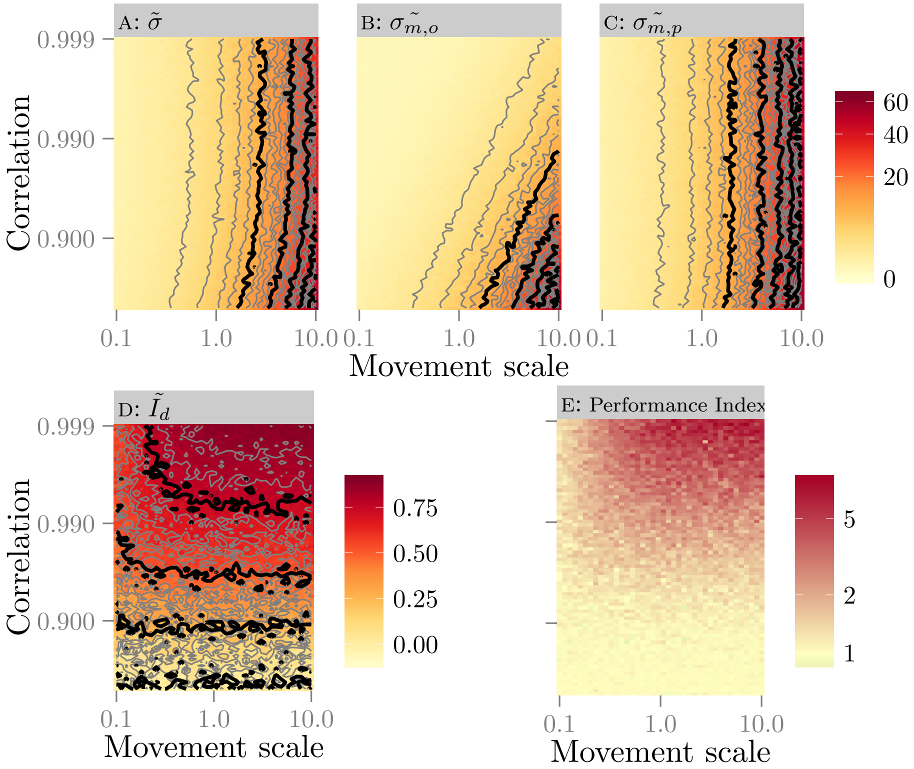

Figure 2: Repetition of the analyses with a higher location error (0.1) showing the effect on the directionality index.
